# RNA-Seq Profiling Shows Divergent Gene Expression Patterns in *Arabidopsis* Grown under Different Densities

**DOI:** 10.3389/fpls.2017.02001

**Published:** 2017-11-28

**Authors:** Di Guo, Xiaoming Song, Min Yuan, Zhenyi Wang, Weina Ge, Li Wang, Jinpeng Wang, Xiyin Wang

**Affiliations:** ^1^School of Life Sciences, North China University of Science and Technology, Tangshan, China; ^2^Center for Genomics and Computational Biology, North China University of Science and Technology, Tangshan, China

**Keywords:** growth density, RNA-Seq, shade avoidance, glutaredoxin, *Arabidopsis*

## Abstract

Plants growing under high-density (HD) conditions experience increased competition for water, nutrients, and light, possibly leading to changes in size, biomass, morphology, and productivity. However, no research has focused on the relationship between whole-genome expression patterns and growth density. Here, we performed whole-genome RNA sequencing to examine the gene expression patterns in *Arabidopsis* grown under low and high densities. Of the 20,660 detected genes, the expression levels of 98 were enhanced and 107 were repressed under HD growth. Further analysis revealed that changes in density influenced metabolism- and stimulus-related genes the most. Furthermore, HD growth led to a shade avoidance phenotype, represented by upward growth and a reduction in rosette leaves. Moreover, a cluster of glutaredoxin genes, *GRXS3, 4, 5, 7*, and *8*, were significantly down-regulated under high density, suggesting that high density affects plant growth mainly by nitrate limitation.

## Introduction

Most plant studies have been conducted using individual potted plants under controlled conditions (Hecht et al., 2016), and much physiological and molecular data have been derived from this. Sowing density is important as plants grown under high-density (HD) conditions compete with each other for water, nutrition, and light, which often leads to changes in plant size, biomass, morphology, and productivity (Hecht et al., 2016). Research about plant density refers to productivity, organ development, nutrition absorption, water heterogeneity, shade avoidance, and flowering (Lemaire et al., 2005; Hagiwara et al., 2010; Li et al., 2011, 2016; El-Zaeddi et al., 2016; Roig-Villanova and Martinez-Garcia, 2016; Song et al., 2016).

Studies on plant density and productivity have mainly focused on crops, vegetables, and medical plants, including wheat, maize, potato, *Brassica*, and *Salvia miltiorrhiza*. People measure the organ size and biomass to determine the optimal sowing density (Li et al., 2011, 2016; Kuai et al., 2015; Liu et al., 2016; Song et al., 2016; Zheng et al., 2016). Besides productivity, plant density also affects organ development (Hecht et al., 2016; Song et al., 2016). The roots of spring barley increased with higher sowing density, particularly in the topsoil. In contrast, the root mass decreased while the stem mass increased with higher sowing density (Hecht et al., 2016). The effects of plant density on maize canopy structure indicated that both lamina width and internode diameter were reduced under high density as a result of a lower growth rate (Song et al., 2016).

Plants compete for nutrition, water and light under HD conditions. Shoot nitrogen (N) dynamics analysis in lucerne showed that N accumulation was less rapid when plant density was high, which was caused by a decrease in leaf biomass (Lemaire et al., 2005). Additionally, plant density influences the volatile compositions of dill, suggesting differences in nutrient absorption at various densities (El-Zaeddi et al., 2016). The effects of water supply heterogeneity on *Perilla frutescens* were greater at higher density than at lower density, indicating that competition for water makes plants more sensitive to water heterogeneity (Hagiwara et al., 2010). To respond the light competition, plants employ two opposing strategies: shade tolerance and shade avoidance. Shade avoidance is associated with a set of responses known as the shade avoidance syndrome (SAS), including stem-like organ elongation, apical dominance, flowering acceleration, branch reduction, and decreasing leaf expansion and yield. Phytochromes, parotid isoelectric focusing (PIF) proteins, HD-ZIP II transcription factors, and auxins were found to be involved in shade avoidance (Morelli and Ruberti, 2002; Ruberti et al., 2012; Gonzalez-Grandio et al., 2013; Roig-Villanova and Martinez-Garcia, 2016).

Flowering is a pivotal event for plants and represents the switch from vegetative to reproductive development. Botto and Coluccio (2007) analyzed the effects of plant density on flowering time in *Arabidopsis* using recombinant inbred lines, and found a variation in flowering time across seasonal and density environments.

Since plant density affects many traits, we want to characterize gene expression under different growth densities. Here, we use RNA sequencing to examine the whole-genome expression patterns of *Arabidopsis* under high and low growth densities to identify density-regulated genes.

## Materials and Methods

### Plant Materials and Growth Conditions

*Arabidopsis* Col-0 was provided by Prof. Ning Li at the Hong Kong University of Science and Technology. The soil used in this study was Klasmann-Deilmann 876. Seeds were first imbibed at 4°C for 3 days before being sowed in the soil. The plants were grown at 22 ± 1°C under continuous white light at an intensity of 100 μmol photons m^-2^ s^-1^. The pots used in this study were 32 × 48 × 9 (depth) cm. The distance between the seeds sown at low density (LD) was 10 cm, while the distance between the seeds in the HD treatment was 2 cm. Three-week-old leaf samples were collected from directly under the white light for angle and diameter measurements, RNA sequencing, and qPCR examination. Plants situated on the edge of the pots were excluded from all experiments. For RNA sequencing, the plants were randomly selected and the rosette leaves of three individual plants were separately collected. More than one leaf was used for the RNA extraction. Leaf samples from different plants were not pooled.

### RNA Extraction and cDNA Preparation

RNA extraction and cDNA preparation kits were provided by Personal Biotechnology Co., Ltd., Shanghai, China. For RNA-Seq analysis, total RNA was extracted using an RNAout kit (TIANDZ, CAT#: 71203). For qPCR, total RNA was extracted using TRIZOL (Invitrogen). mRNA was purified and cDNA was prepared using the Truseq Stranded mRNA LT Sample Prep Kit (Illumina).

### RNA Sequencing

RNA sequencing services were provided by Personal Biotechnology Co., Ltd., Shanghai, China. The constructed cDNA libraries were examined using an Agilent High Sensitivity DNA Kit, and the average fragment length was 250 bp. The libraries were sequenced using Illumina NextSeq500 to generate paired-end reads of 150 bp. The sequenced raw reads were processed to obtain clean reads using the following strategy: (i) remove reads with adaptor contamination; (ii) remove low quality reads whose average quality was less than Q20; and (iii) remove the reads with a final length of less than 50 bp. The quality of the clean reads was assessed using FastQC^1^ (**Supplementary Figure S1**). Three replicates of each treatment were sequenced and the raw data were uploaded to NCBI and deposited in the sequence read archive (SRA^2^). The biosample accession isSAMN07267285 and the corresponding address is: https://www.ncbi.nlm.nih.gov/sra/?term=SAMN07267285.

High-quality clean reads were used for further analysis. Bowtie2 and TopHat2^3^ were used for mapping, and the reference genome was Arabidopsis_thaliana.TAIR10.28.dna.toplevel.fa. Genome annotation was based on Ensembl^4^ and KEGG (Kyoto Encyclopedia of Genes and Genomes^5^). Gene expression analysis was performed using HTSeq^6^ and DESeq^7^ at different expression levels (**Supplementary Table S6**). Up- and down-regulation of genes was considered to have occurred when the *P*-value was <0.05 and the absolute fold changes were ≥2.0. Gene ontology (GO) and KEGG orthology (KO) analysis of the genes with differential expression was also performed (**Supplementary Figure S4** and **Supplementary Tables S7, S8**).

### qPCR Analysis

The qPCR conditions were set as follows: 95°C for 5 min, followed by 40 cycles of 95°C at 15 s and 60°C at 30 s. *ACT2* was used as an internal control. Primers used in the qPCR are shown in **Supplementary Table S9**, and were assessed via a standard curve. The amplification and melting curves are shown in **Supplementary Figure S5**. The values were reported using the 2^ΔΔC_T_^ method. A *P*-value < 0.05 and absolute fold change ≥2.0 signified differential expression.

### Nitrate Content Determination

Nitrate determination was performed according to an approach in a previous report (Lv et al., 2004). Three-week rosette leaves were collected, dried, and digested using the Kjeldathl method with H_2_SO_4_ and H_2_O_2_. Nitrate concentration was measured at OD_210_. KNO_3_ was used for drawing the standard curve. The formula was *A* = 7.5857*C_NO3_-* – 0.0466. *r* = 0.9970.

### Gene Name and Corresponding Locus in This Study

*GRXS3*: At4g15700; *GRXS4*: At4g15680; *GRXS5*: At4g15690; *GRXS7*: At4g15670; *GRXS8*: At4g15660; *FD*: At4g35900; *FT*: At1g65480; *FUL*: At5g60910; *AtHB-2*: At4g16780; *HFR1*: At1g02340; *PIL1*: At2g46970; *ACT2*: At3g18780.

## Results

### RNA Sequencing of *Arabidopsis* under Different Growth Densities

RNA sequencing was used to examine the gene expression patterns in *Arabidopsis* under low and high growth densities. For each treatment, three replicates were sequenced using Illumina NextSeq500. Approximately 30 million reads of raw tags were obtained for each sample (**Supplementary Table S1**), with the GC distribution close to theoretical distribution (**Supplementary Figure S2**). After filtering with stringent criteria (see section Materials and Methods), we finally obtained 31.26, 32.18, and 28.70 million reads for the high growth density samples, and 31.98, 30.10, and 31.56 million reads for the low growth density samples (**Supplementary Table S2**). All raw data were uploaded onto NCBI SRA and the biosample accession is SAMN07267285. The Pearson’s correlation coefficients of the three replicates for each density were larger than 80%, suggesting appreciable correlation between them (**Supplementary Figure S3**). A percentage of 90% of the reads were mapped onto the *Arabidopsis* genome, most of which were uniquely mapped (**Supplementary Table S3**). Among those genome-mapped reads, about 90% were mapped to gene region, and more than 99% were mapped onto exons (**Supplementary Table S4**). In total, 20,660 sequenced genes were recovered and were distributed in five nuclear chromosomes (**Supplementary Table S5**).

### Gene Expression of *Arabidopsis* under Different Growth Densities

Among the 20,660 genes, the expression levels of 20,455 were not influenced by growth density, and only 205 constituted differentially expressed genes (DEGs) under the different growth densities (**Supplementary Table S6**). Under high growth density, 98 genes were up-regulated and 107 genes were down-regulated in comparison with the LD conditions (**Supplementary Figure S4A** and **Supplementary Table S6**).

We classified the density-related genes by GO enrichment analysis. Biological processes, metabolic processes, cellular processes, death, stimulus, and stress obtained *P*-values < 0.01 and were thus likely related to changes in plant density (**Supplementary Figure S4B** and **Supplementary Table S7**). Cellular components, nucleus, endoplasmic reticulum, external encapsulating structure, and extracellular region were significantly related to changes in density, and the corresponding *P*-values for external encapsulating structure and extracellular region were <0.01 (**Supplementary Figure S4B** and **Supplementary Table S7**). With regards to molecular functions, the most changed DEGs were detected in the binding group, indicating significant change with *P*-values < 0.05 (**Supplementary Figure S4B** and **Supplementary Table S7**).

The KEGG enrichment analysis allowed us to divide all the genes into six categories, including metabolism, genetic information processing, environmental information processing, cellular processes, organismal systems, and human diseases (**Supplementary Table S8**). We discovered that growth density was the most influential metabolism category, among which amino acid metabolism, secondary metabolite biosynthesis, and xenobiotic biodegradation were significantly affected (**Supplementary Figure S4C** and **Supplementary Table S8**). In addition, the DNA replication and repair pathway in genetic information processing was influenced by high growth density with *P* < 0.05 (**Supplementary Table S8**). Generally, metabolism was influenced more than genetic functioning based on the significantly smaller *P*-values (**Supplementary Figure S4C** and **Supplementary Table S8**). The other four categories did not exhibit much change between the high and low growth densities (**Supplementary Table S8**).

### High Density Causes a Shade Avoidance Response Phenotype

**FIGURE 1:**
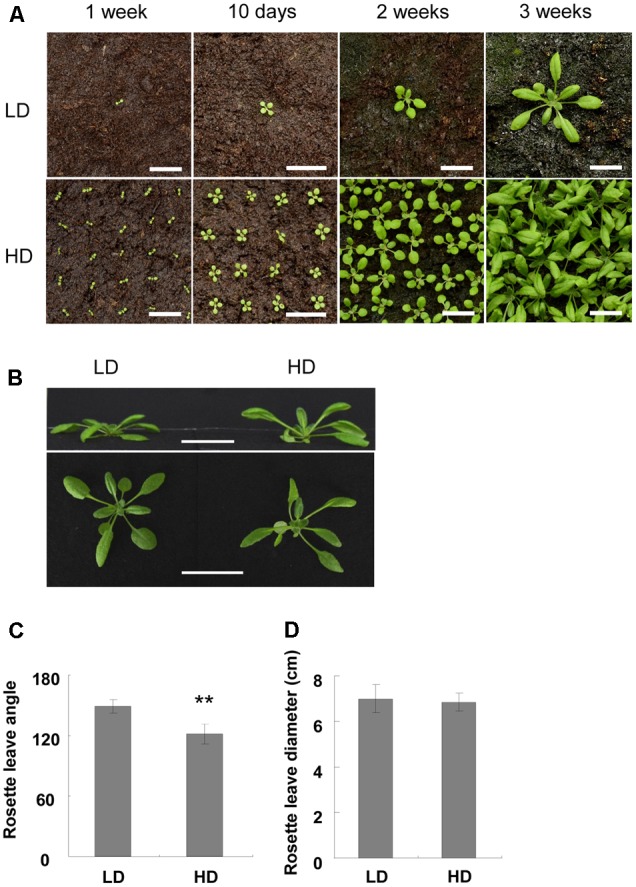
Characterization of Col-0 at high and low densities. **(A)** Photo of Col-0 at different stages and densities. Bars = 2 cm. **(B)** Side view of 3-week-old plants in different growth densities. Bars = 3 cm. **(C,D)** Rosette leaf angle **(C)** and rosette diameter **(D)** of 3-week-old plants. ^∗∗^Represents *P*-value < 0.01.

In our study, we found that plants grown under high density displayed a shade avoidance response, which was represented by a smaller rosette leaf angle and reduction in rosette leaves. The leaves of Col-0 began to reach the proximity of the other plants at about 2 weeks (**Figure 1A**). After that, they grew upward to avoid the shade. The rosette leaf angle thus became smaller compared with the low-density plants (**Figures 1B,C**). However, the rosette leaf diameter was similar between the two treatments (**Figure 1D**).

**FIGURE 2:**
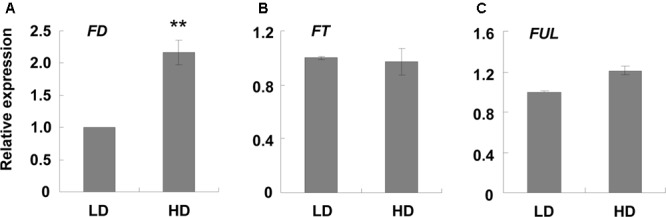
qPCR analysis of some flowering-related genes at different densities. Three-week-old leaf samples under high and low growth densities were collected. qPCR was performed to examine the expression levels of **(A–C)** *FD, FT*, and *FUL*. *ACT2* was used as an internal control. LD, low density; HD, high density. ^∗∗^Represents *P*-value < 0.01 and the fold changes > 2.

Another shade avoidance response is the reduction in rosette leaves at bolting time. Although there was variation between the different sets of experiments, the bolting time of the rosette leaves of the HD plants was 2 less than those grown at LD (**Table 1**). However, flowering time was similar between the high and low densities (**Table 1**). As rosette leaf number is an important indicator of flowering time (Abe et al., 2005; Wigge et al., 2005), we searched for flowering-related genes and found that only *FD* was up-regulated under high density (**Supplementary Table S6**). qPCR validation demonstrated that *FD* was induced about 2-fold when the plants were grown at high density (**Figure 2A**). It was reported that the combination of FD with FT stimulates flowering, and the over-expression of *FD* induces *FUL* expression (Wigge et al., 2005; Amasino, 2010; Srikanth and Schmid, 2011). Here, we assessed the expression levels of *FT* and *FUL* under different growth densities and discovered that neither *FT* nor *FUL* showed any difference between high and low growth densities in both the RNA-Seq and qPCR analysis (**Figures 2B,C** and **Supplementary Table S6**).

**Table 1:** Bolting time and rosette leaf number of Col-0 under different growth conditions.

	Experiment 1			Experiment 2		
	Bolting time	Rosette leaf	*N*	Bolting time	Rosette leaf	*N*
	(day)	number		(day)	number	
Low density	26.5 ± 2.2	14.8 ± 1.6 (12–18)	28	36.8 ± 3.0	21.0 ± 2.1 (18–25)	25
High density	25.4 ± 1.7	12.8 ± 1.1 (11–15)	97	36.9 ± 1.5	18.7 ± 1.1 (17–21)	79

**FIGURE 3:**
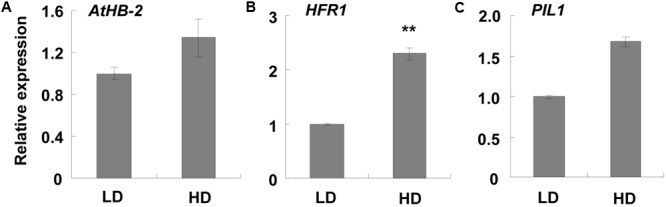
qPCR analysis of SAS-related genes at different densities. Three-week-old leaf samples grown under high and low densities were collected. qPCR was performed to examine the expression levels of **(A–C)** *AtHB-2, HFR1*, and *PIL1*. *ACT2* was used as an internal control. LD, low density; HD, high density. ^∗∗^Represents *P*-value < 0.01 and the fold changes > 2.

Since a shade avoidance response phenotype was detected, we further assessed any changes in SAS-related genes. We found that marker genes, such as *ATHB-2, HFR1*, and *PIL1*, did not show any obvious differences in the RNA-Seq analysis (**Supplementary Table S6**). However, qPCR analysis indicated that they were induced about 1.34, 2.30, and 1.68-fold, respectively (**Figure 3**), indicating that shade avoidance was slightly induced.

### High Density Repressed the *GRXS3*/*4*/*5*/*7*/*8* Cluster Genes

**FIGURE 4:**
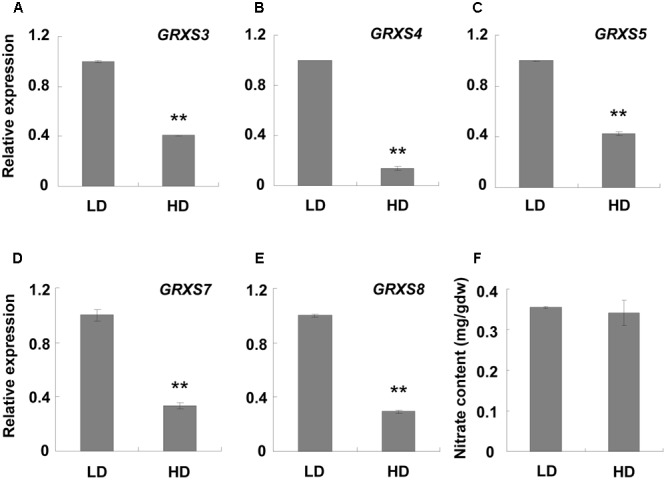
qPCR analysis of *GRXS* genes and rosette leaf nitrate content at different densities. Three-week-old leaf samples under high and low growth densities were collected. **(A–E)** qPCR was performed to examine the expression levels of the *GRXS3, 4, 5, 7*, and *8* genes. *ACT2* was used as an internal control. ^∗∗^Represents *P*-value < 0.01 and the fold change > 2. **(F)** Nitrate content in the rosette leaves was measured. LD, low density; HD, high density; mg/gdw, mg per gram dry weight.

The RNA sequencing results showed that a cluster of *GRXS* genes on chromosome 4, named *GRXS3, 4, 5, 7*, and *8*, were significantly down-regulated under high density (**Supplementary Table S6**). qPCR validation demonstrated that all these genes were down-regulated at least two-fold under high density conditions in comparison to LD conditions (**Figures 4A–E**). Since this *GRXS* cluster was previously reported to be closely associated with soil nitrates (Patterson et al., 2016; Walters and Escobar, 2016), the nitrate content of the rosette leaves was assessed. However, the nitrate content was similar between plants grown under high and low densities (**Figure 4F**).

### High Density Does Not Induce an Abiotic Stress Response

Plants growing under high density compete with each other for water, light, and nutrients, which often leads to changes in size, biomass, morphology, and productivity (Hecht et al., 2016). Thus, extreme high density constitutes an abiotic stress. A previous study showed that abscisic acid (ABA) was responsible for plant defense against abiotic stresses, while ethylene (ET), salicylic acid (SA), and jasmonate (JA) mainly responded to biotic stresses (Verma et al., 2016). Here, we assessed the expression patterns of some ABA biosynthesis and response genes, such as *ABA1, AAO3, NCED3, ABA3, ABF2, ABF3*, and *ABF4* (Verma et al., 2016). However, no significant changes were observed in the expression of all these genes between the high and low densities (**Supplementary Table S6**).

## Discussion

### Growth Density Caused Variation in Global Gene Expression

We found that relatively few (1%, 205/20,660) of the considered *Arabidopsis* genes were differentially expressed under the different planting densities (**Supplementary Table S6**). GO enrichment analysis suggested that density obviously influences metabolic processes (**Supplementary Figure S4B** and **Supplementary Table S7**), which is consistent with the findings of the KEGG analysis whereby three metabolic pathways were significantly affected (**Supplementary Figure S4C** and **Supplementary Table S8**). Since density constitutes an environmental factor, stimulus- and stress-related genes were greatly affected (**Supplementary Figure S4B** and **Supplementary Table S7**), and all *P*-values were < 0.01, implying interaction between these genes and the changing environment. By performing cellular component analysis, we found that genes in the extracellular region, particularly the external encapsulating structure, changed the most (*P*-values < 0.01, **Supplementary Figure S4B** and **Supplementary Table S7**). This group of genes is located in the primary cell wall, implying that density influences cell wall development.

### High Density Led to a Shade Avoidance Phenotype

Plants exhibit shade avoidance when grown at high density as the proximity to other vegetation results in competition for light. However, most SAS-related genes did not show much change in this study (**Supplementary Table S6**), although a shade avoidance phenotype was observed. This may be the result of the growth conditions set in this study. Previous studies reduced both the ratio and intensity of R/FR light to induce a shade avoidance response (Sessa et al., 2005; Ciolfi et al., 2013). This treatment ensures that the entire plant is under low light and cannot escape the shade, which often induces a quick and obvious shade avoidance response. In our study, continuous white light was used and the distance between the *Arabidopsis* seeds in the HD treatment was 2 cm. In the first 2 weeks, the seedlings did not crowd each other, which allowed each plant to get enough light. Therefore, no shade avoidance was induced at this time. When the plants became larger, they started touching at their proximity at about 2 weeks (**Figure 1A**). Shade avoidance was then induced and some response genes were up- or down- regulated. After that, the rosette leaves changed the growth direction to avoid the shade. When the rosette leaves were growing upward, 2 cm offered enough space for the plants to avoid the shade and obtain adequate light. Since the shade avoidance response is rapid and reversible (Morelli and Ruberti, 2002), the plants then stopped the shade avoidance response and those up- or down- regulated genes restored to normal levels. As we collected the samples at 3 weeks, the additional 1-week was sufficient for the restoration of the SAS response. This may explain why the induction of SAS-related genes was relatively low (∼2-fold) in this study (**Figure 3**).

Another SAS response was the reduction in rosette leaves at bolting time (**Table 1**). The rosette leaf number and bolting time are two indicators of flowering time (Pouteau et al., 2004; Abe et al., 2005; Wigge et al., 2005). In most cases, a reduction in rosette leaves is accompanied by earlier bolting (Pouteau et al., 2004). However, in our study there was no difference in bolting time, and only a reduction in rosette leaves was observed (**Table 1**). Upon evaluating the RNA-Seq data, we found that only one flowering-related gene, *FD*, was up-regulated (**Supplementary Table S6**). qPCR validation demonstrated that the induction of *FD* was about 2-fold (**Figure 2A**). We speculated that this phenotype was caused by the low induction of *FD*. Since SAS caused earlier flowering, we hypothesized that when the plants began to encroach on each other, shade avoidance occurred and *FD* was up-regulated. Therefore, the plants successfully avoided the shade and stopped the SAS response, resulting in a low induction of *FD* (∼2-fold).

It was also reported that the over-expression of *FD* by the *35S* promoter caused obvious earlier flowering, representing by 5 fewer rosette leaves than the wild-type at bolting time (Wigge et al., 2005). Compared with the constitutive promoter *35S*, the induction fold of *FD* was very low (∼2-fold) in our study (**Figure 2A**). This low induction is not enough to cause the obvious flowering phenotype. In our study, only 2 fewer rosette leaves were observed, and no earlier bolting was noted. In addition, this low *FD* induction was not enough to induce other related genes, such as *FUL* and *FT*. The overexpression of *FD* by the *35S* promoter resulted in an enhanced expression level of *FUL* in the leaves (Wigge et al., 2005). *FUL* was only induced 1.2-fold in our results (**Figure 2C** and **Supplementary Table S6**). Although FD, in combination with FT, was considered to stimulate plant flowering (Abe et al., 2005), the expression level of *FT* in our study did not differ under the different densities (**Figure 2B**). Taken together, we suggest that the reduction in rosette leaves was caused by enhanced *FD* expression.

A similar phenotype was reported previously (Botto and Coluccio, 2007). For the *Arabidopsis ‘*Bay’ ecotype, bolting time was similar between the high and low densities, but three fewer rosette leaves at bolting time were observed at high density than at LD (Botto and Coluccio, 2007). In their study, 8 seeds per pot (8 cm in diameter) constituted the HD treatment, which is similar to our conditions (2 cm distance). We speculated that the flowering phenotype in their study was also caused by SAS and induction of the *FD* gene.

### Plants under High Density Compete for Nitrate Absorption

In our study, we found that *GRXS3, 4, 5, 7*, and *8* were significantly repressed under high density (**Figure 4**). Previous literature reported that the *GRXS3, 4, 5, 7*, and *8* genes belong to class III glutaredoxins and have high similarity in both RNA and protein sequences (Gutsche et al., 2015; Walters and Escobar, 2016). Silencing *GRXS3* led to the reduced expression of *GRXS4, 5, 7*, and *8*, and a longer primary root compared with the control group, suggesting that they acted as negative regulators of primary root growth (Patterson et al., 2016; Walters and Escobar, 2016). Therefore, we presumed that *Arabidopsis* grown at high density possessed longer roots compared with that at LD, since the expression of *GRXS3, 4, 5, 7*, and *8* was obviously down-regulated. At this stage we must note that we failed to retrieve root length data because it was difficult to separate the roots from the soil, especially in the HD treatment. However, a previous study reported that root length in barley increased with increased sowing density, especially in the topsoil (Hecht et al., 2016). Combined with our results, this increase might also be caused by the depression of *GRXS* genes.

*GRXS* genes are particularly sensitive to nitrate, but not ammonia. Nitrate treatment causes an obvious accumulation of *GRXS* genes and an inhibitory effect on primary roots. Silencing the *GRXS3, 4, 5, 7*, and *8* genes resulted in a longer primary root compared with the control group, implying that *GRXS* genes mediated the inhibitory effect of nitrate on primary root growth (Patterson et al., 2016; Walters and Escobar, 2016). When plant roots are grown in nitrate-rich regions of the soil, the accumulated nitrate induces glutaredoxins, which suppress primary root growth. Lateral roots are also induced by soil nitrate, which enhance the root absorption ability (Zhang and Forde, 1998; Patterson et al., 2016; Walters and Escobar, 2016). Considering our data, we hypothesized that plants at LD do not need to compete for nutrients, and the adequate nitrate would induce the *GRXS* genes and suppress primary root growth. In contrast, plants at high density compete with each other for soil nutrients, including nitrate, which leads to a surrounding nitrate deficiency. This deficiency will cause a reduction in *GRXS* genes, which results in a longer primary root. The longer root helps the plants to reach more nitrates in the soil. This explains why nitrate accumulation was similar between the different densities (**Figure 4F**). This mechanism helps plants to compete for nutrition and survive when growing under high density.

Based on our RNA-Seq analysis, we did not find significant expression changes in other nutrition-related genes between the two growth densities, including ammonia (**Supplementary Table S6**). This result indicated that nitrate is the major limiting factor in agricultural productivity when plants are grown under high density. This is not surprising as nitrate is the most-used fertilizer in agriculture as a source of nitrogen compared with ammonium and urea (Noguero and Lacombe, 2016). In addition, some literature has reported that cabbage prefers nitrate to ammonium as a nitrogen source (Tian and Li, 2000; Zhang and Wei, 2002). Since both *Arabidopsis* and cabbage belong to Brassicaceae, nitrate should also be the primary nitrogen source of *Arabidopsis*. That explains the obvious down-regulation of glutaredoxins, which are especially sensitive to nitrate.

### High Density Does Not Cause a Stress Effect When There Is Adequate Water, Nutrition, and Light

High density did not cause an abiotic stress response, and similar expression levels of ABA biosynthesis and response genes were observed (**Supplementary Table S6**). In addition, the RNA-Seq samples of the different densities were highly correlated (**Supplementary Figure S3**), indicating that growth density did not cause any serious global changes in gene expression. The possible reason for the phenotype is that although it was a HD treatment, the water, nutrition, and light were adequate. Therefore, we conclude that under conditions of adequate light, water, and nutrition, density itself does not significantly affect plant growth in *Arabidopsis*.

## Author Contributions

XW conceived and led the research. DG and XS performed all the experiments and data analysis. XW, DG, and XS wrote the paper. MY, ZW, WG, LW, and JW participated in data discussion and manuscript revision.

## Conflict of Interest Statement

The authors declare that the research was conducted in the absence of any commercial or financial relationships that could be construed as a potential conflict of interest.

## Footnotes

1 http://www.bioinformatics.babraham.ac.uk/projects/fastqc/

2 https://www.ncbi.nlm.nih.gov/sra

3 http://tophat.cbcb.umd.edu/

4 http://ensemblgenomes.org

5 http://www.genome.jp/kegg/

6 http://htseq.readthedocs.io/en/release_0.9.1/

7 http://www.bioconductor.org/packages/release/bioc/html/DESeq2.html

## Supplementary Material

FIGURE S1 Clean reads quality analysis using FastQC. Q > 28 is considered to be high quality and is labeled with a green background.

FIGURE S2 GC content distribution.

FIGURE S3 Pearson correlation coefficient analysis of three replicates for each treatment. The correlation coefficients were calculated using log2 (FPKM). LD, low density; HD, high density.

FIGURE S4 Characterization of DEGs under high and low densities. **(A)** DEGs under high and low densities. HD represents high density. **(B)** GO enrichment analysis of DEGs. Biological process, cellular component, and molecular function-related genes were identified. Lines represent *P*-value = 0.05. **(C)** KEGG enrichment analysis of metabolism-related DEGs. Lines represent *P*-value = 0.05.

FIGURE S5 Melting curve and amplification plot of all qPCR results.

TABLE S1 Summary of the sequencing and assembly.

TABLE S2 Summary of the sequencing data filtered by Q20.

TABLE S3 Mapped results of the RNA sequencing data.

TABLE S4 Detailed analysis of the gene map counts.

TABLE S5 Chromosome distribution of sequenced genes.

TABLE S6 Differentially and non-differentially expressed genes under the different growth densities.

TABLE S7 GO enrichment analysis of differentially expressed genes.

TABLE S8 KEGG enrichment analysis of differentially expressed genes.

TABLE S9 qPCR primers used in this study.

## References

[B1] Abe M. Kobayashi Y. Yamamoto S. Daimon T. Yamaguchi A. Ikeda Y. (2005). FD, a bZIP protein mediating signals from the floral pathway integrator FT at the shoot apex. *Science* 309 1052–1056. 10.1126/science.1115983 16099979

[B2] Amasino R. (2010). Seasonal and developmental timing of flowering. *Plant J.* 61 1001–1013. 10.1111/j.1365-313X.2010.04148.x 20409274

[B3] Botto J. Coluccio M. P. (2007). Seasonal and plant-density dependency for quantitative trait loci affecting flowering time in multiple populations of *Arabidopsis thaliana*. *Plant Cell Environ.* 30 1465–1479. 10.1111/j.1365-3040.2007.01722.x 17897416

[B4] Ciolfi A. Sessa G. Sassi M. Possenti M. Salvucci S. Carabelli M. (2013). Dynamics of the shade-avoidance response in *Arabidopsis*. *Plant Physiol.* 163 331–353. 10.1104/pp.113.221549 23893169 PMC3762654

[B5] El-Zaeddi H. Martinez-Tome J. Calin-Sanchez A. Burlo F. Carbonell-Barrachina A. A. (2016). Irrigation dose and plant density affect the volatile composition and sensory quality of dill (*Anethum graveolens* L.). *J. Sci. Food Agric.* 97 427–433. 10.1002/jsfa.7890 27392118

[B6] Gonzalez-Grandio E. Poza-Carrion C. Sorzano C. O. S. Cubas P. (2013). *BRANCHED1* promotes axillary bud dormancy in response to shade in *Arabidopsis*. *Plant Cell* 25 834–850. 10.1105/tpc.112.108480 23524661 PMC3634692

[B7] Gutsche N. Thurow C. Zachgo S. Gatz C. (2015). Plant-specific CC-type glutaredoxins: functions in developmental process and stress response. *J. Biol. Chem.* 396 495–509. 10.1515/hsz-2014-0300 25781542

[B8] Hagiwara Y. Kachi N. Suzuki J. (2010). Effects of temporal heterogeneity of water supply on the growth of *Perilla frutescens* depend on plant density. *Ann. Bot.* 106 173–181. 10.1093/aob/mcq096 20495200 PMC2889804

[B9] Hecht V. L. Temperton V. M. Nagel K. A. Rascher U. Postma J. A. (2016). Sowing density: a neglected factor fundamentally affecting root distribution and biomass allocation of field grown spring barley (*Hordeum vulgare* L.). *Front. Plant Sci.* 7:944. 10.3389/fpls.2016.00944 27446171 PMC4923255

[B10] Kuai J. Sun Y. Zuo Q. Huang H. Liao Q. Wu C. (2015). The yield of mechanically harvested rapeseed (*Brassica napus L*.) can be increased by optimum plant density and row spacing. *Sci. Rep.* 5:18835. 10.1038/srep18835 26686007 PMC4685391

[B11] Lemaire G. Avice J. Kim T. Ourry A. (2005). Developmental changes in shoot N dynamics of Lucerne (*Medicago sativa* L.) in relation to leaf growth dynamics as a function of plant density and hierarchical position within the canopy. *J. Exp. Bot.* 56 935–943. 10.1093/jxb/eri084 15710638

[B12] Li C. G. Sheng S. J. Pang E. C. K. May B. Xue C. C. L. (2011). Plant density-dependent variations in bioactive markers and root yield in Australian-grown *Salvia miltiorrhiza* Bunge . *Chem. Biodiver.* 8 699–709. 10.1002/cbdv.201000192 21480516

[B13] Li Y. Cui Z. Ni Y. Zheng M. Yang D. Jin M. (2016). Plant density effect on grain number and weight of two winter wheat cultivars at different spikelet and grain positions. *PLOS ONE* 11:e0155351. 10.1371/journal.pone.0155351 27171343 PMC4865215

[B14] Liu T. Wang Z. Cai T. (2016). Canopy apparent photosynthetic characteristics and yield of two spike-type wheat cultivars in response to row spacing under high plant density. *PLOS ONE* 11:e0148582. 10.1371/journal.pone.0148582 26845330 PMC4741391

[B15] Lv W. Ge Y. Wu J. Chang J. (2004). Study on the method for the determination of nitric nitrogen, ammoniacal nitrogen and total nitrogen in plant. *Spectrosc. Spectral Anal.* 24 204–206. 15769018

[B16] Morelli G. Ruberti I. (2002). Light and shade in the photocontrol of *Arabidopsis* growth. *Trends Plant Sci.* 7 399–404. 10.1016/S1360-1385(02)02314-2 12234731

[B17] Noguero M. Lacombe B. (2016). Transporters involved in root nitrate uptake and sensing by *Arabidopsis*. *Front. Plant Sci.* 7:1391. 10.3389/fpls.2016.01391 27708653 PMC5030233

[B18] Patterson K. Walters L. A. Cooper A. M. Olvera J. G. Rosas M. A. Rasmusson A. G. (2016). Nitrate-regulated glutaredoxins control *Arabidopsis thaliana* primary root growth. *Plant Physiol.* 170 989–999. 10.1104/pp.15.01776 26662603 PMC4734588

[B19] Pouteau S. Ferret V. Gaudin V. Lefebvre D. Sabar M. Zhao G. (2004). Extensive phenotypic variation in early flowering mutants of *Arabidopsis*. *Plant Physiol.* 135 201–211. 10.1104/pp.104.039453 15122022 PMC429349

[B20] Roig-Villanova I. Martinez-Garcia J. F. (2016). Plant responses to vegetation proximity: a whole life avoiding shade. *Front. Plant Sci.* 7:236. 10.3389/fpls.2016.00236 26973679 PMC4770057

[B21] Ruberti I. Sessa G. Ciolfi A. Possenti M. Carabelli M. Morelli G. (2012). Plant adaptation to dynamically changing environment: the shade avoidance response. *Biotechnol. Adv.* 30 1047–1058. 10.1016/j.biotechadv.2011.08.014 21888962

[B22] Sessa G. Carabelli M. Sassi M. Ciolfi A. Possenti M. Mittempergher F. (2005). A dynamic balance between gene activation and repression regulates the shade avoidance response in *Arabidopsis.* *Genes Dev.* 19 2811–2815. 10.1101/gad.364005 16322556 PMC1315388

[B23] Song Y. Rui Y. Bedane G. Li J. (2016). Morphological characteristics of maize canopy development as affected by increased plant density. *PLOS ONE* 11:e0154084. 10.1371/journal.pone.0154084 27129101 PMC4851380

[B24] Srikanth A. Schmid M. (2011). Regulation of flowering time: all roads lead to Rome. *Cell. Mol. Life Sci.* 68 2013–2037. 10.1007/s00018-011-0673-y 21611891 PMC11115107

[B25] Tian H. Li S. (2000). Uptake capacity of several vegetable crops to nitrate and ammonium. *Plant Nutr. Fertil. Sci.* 6 194–201.

[B26] Verma V. Ravindran P. Kumar P. P. (2016). Plant hormone-mediated regulation of stress responses. *BMC Plant Biol.* 16:86. 10.1186/s12870-016-0771-y 27079791 PMC4831116

[B27] Walters L. A. Escobar M. A. (2016). The *AtGRXS3/4/5/7/8* glutaredoxin gene cluster on *Arabidopsis thaliana* chromosome 4 is coordinately regulated by nitrate and appears to control primary root growth. *Plant Signal. Behav.* 11:e1171450. 10.1080/15592324.2016.1171450 27049601 PMC4883855

[B28] Wigge P. A. Kim M. C. Jaeger K. E. Busch W. Schmid M. Lohmann J. U. (2005). Integration of spatial and temporal information during floral induction in *Arabidopsis*. *Science* 309 1056–1059. 10.1126/science.1114358 16099980

[B29] Zhang H. Forde B. G. (1998). An *Arabidopsis* MADS box gene that controls nutrient-induced changes in root architecture. *Science* 279 407–409. 10.1126/science.279.5349.407 9430595

[B30] Zhang S. Wei X. (2002). A comparative study of vegetables’ absorption of NO3^-^-N and NH4^+^-N. *J. Lanzhou Univ. Nat. Sci.* 38 77–84.

[B31] Zheng S. Wang L. Wan N. Zhong L. Zhou S. He W. (2016). Response of potato tuber number and spatial distribution to plant density in different growing seasons in Southwest China. *Front. Plant Sci.* 7:365. 10.3389/fpls.2016.00365 27092146 PMC4824783

