# Supplementary figures and images for: RNA-Seq Profiling Shows Divergent Gene Expression Patterns in Arabidopsis Grown under Different Densities
Source: Front Plant Sci. 2017 Nov 28;8:2001. doi: 10.3389/fpls.2017.02001 (PMC5712407; doi:10.3389/fpls.2017.02001)

**FIGURE S1**

**FIGURE S2**

**FIGURE S3**

**
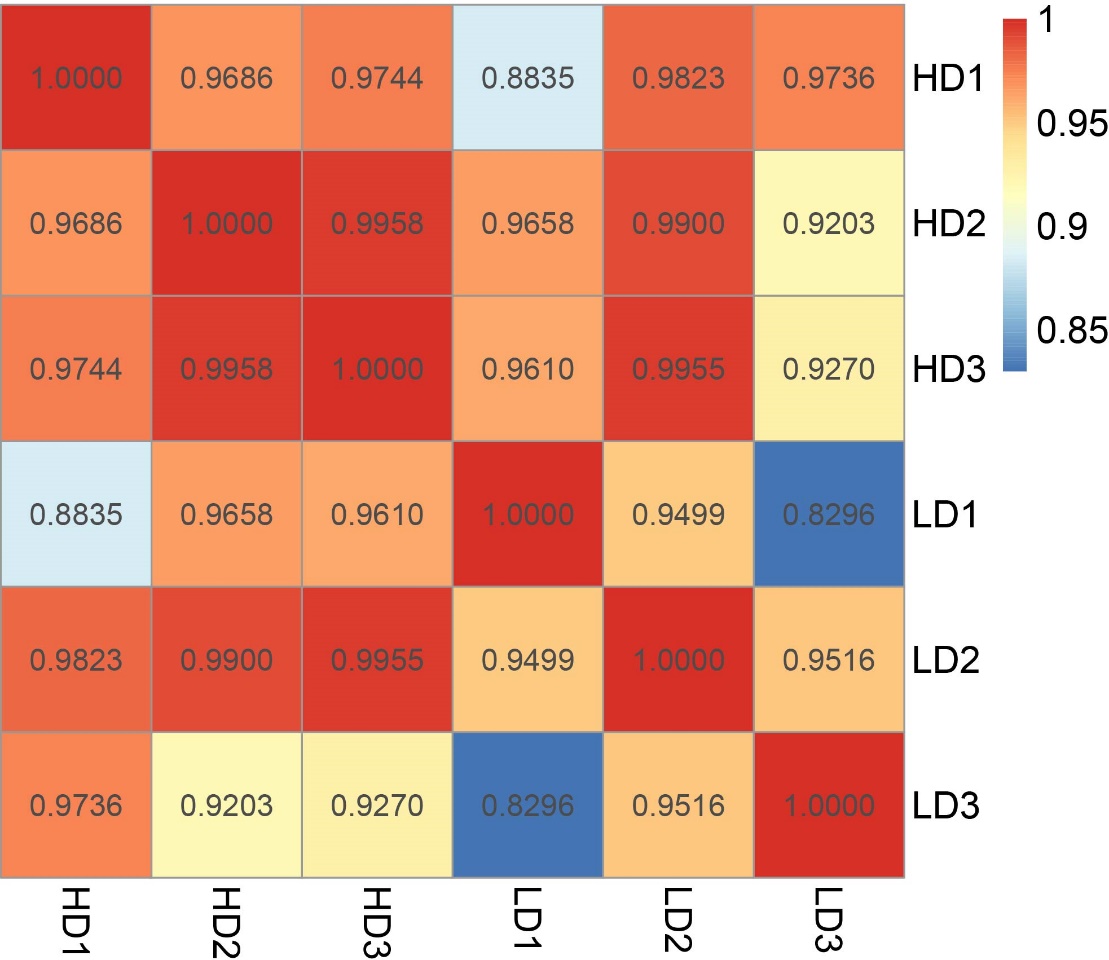
**

**FIGURE S4**

Supplement: FIGURE S1 — Clean reads quality analysis using FastQC. Q > 28 is considered to be high quality and is labeled with a green background. [file Figures_1-4.docx]
