# Supplementary material for: RNA-Seq Profiling Shows Divergent Gene Expression Patterns in Arabidopsis Grown under Different Densities
Source: Front Plant Sci. 2017 Nov 28;8:2001. doi: 10.3389/fpls.2017.02001 (PMC5712407; doi:10.3389/fpls.2017.02001)

Figure S5

ACT2 (At3g18780)

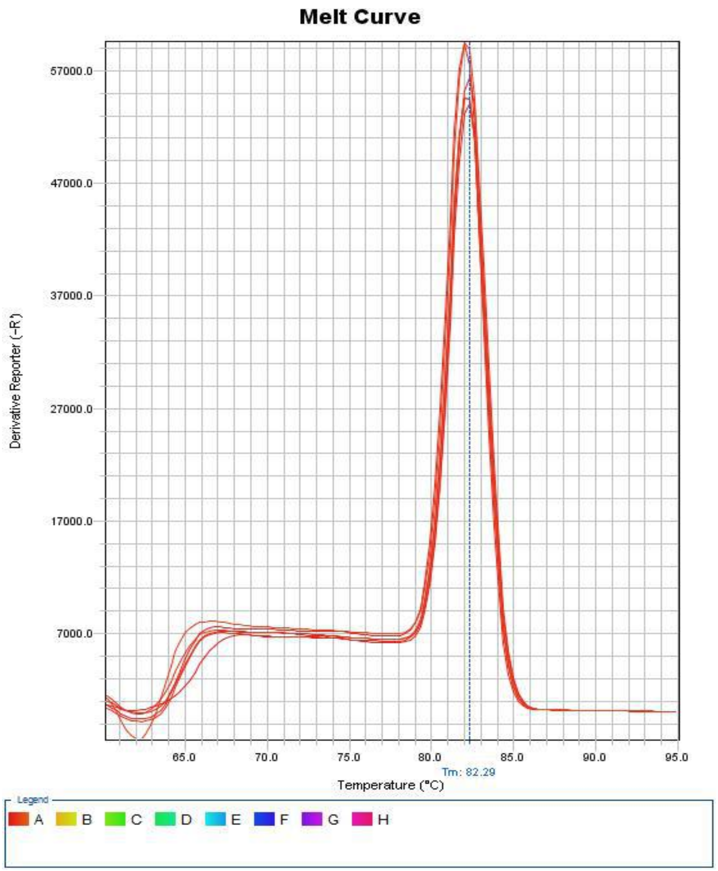

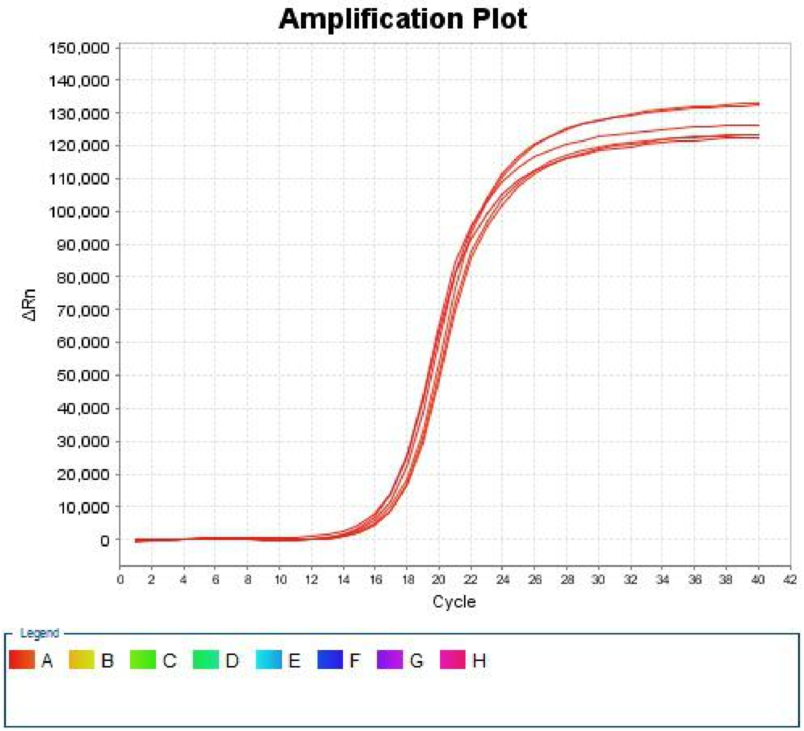

Figure S5

GRXS3 **(At4g15700)**

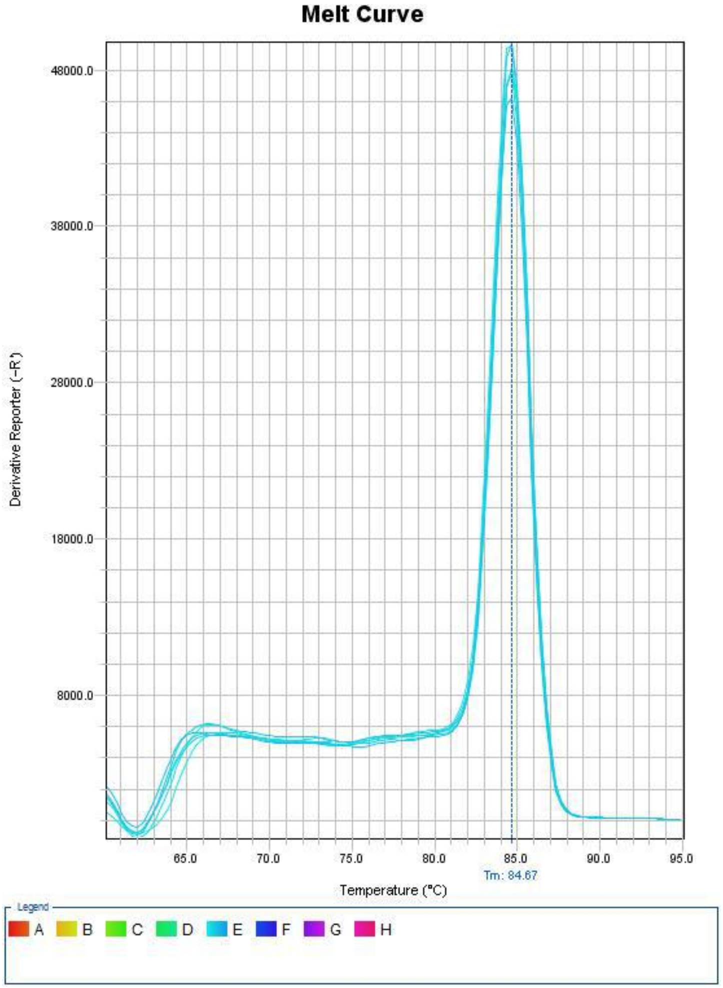

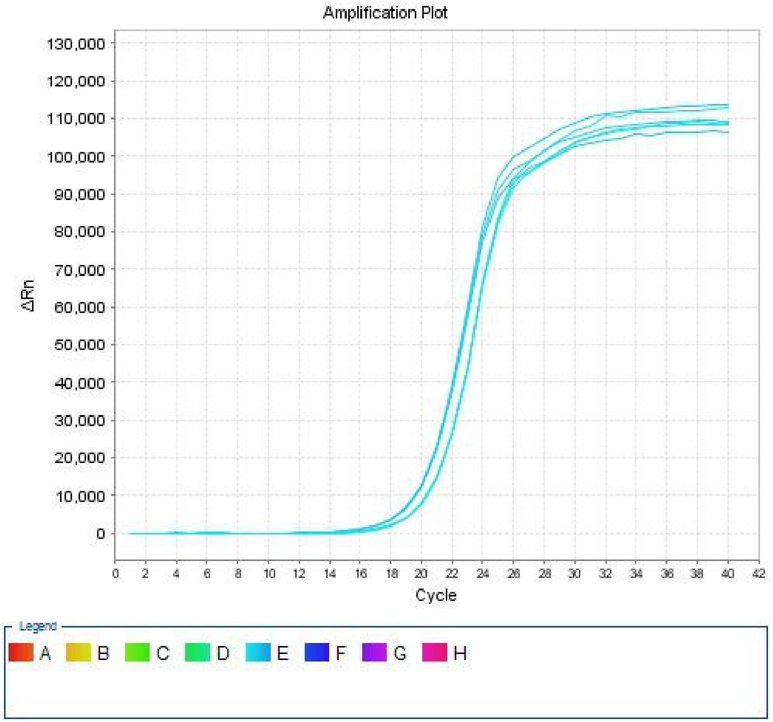

Figure S5

GRXS4 **(At4g15680)**

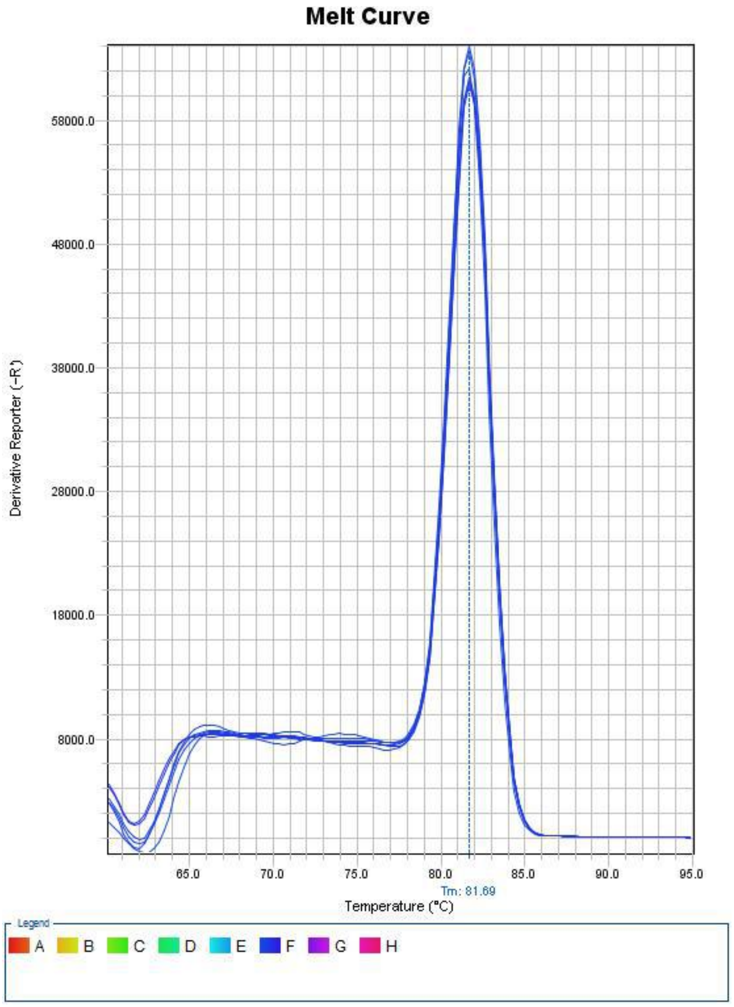

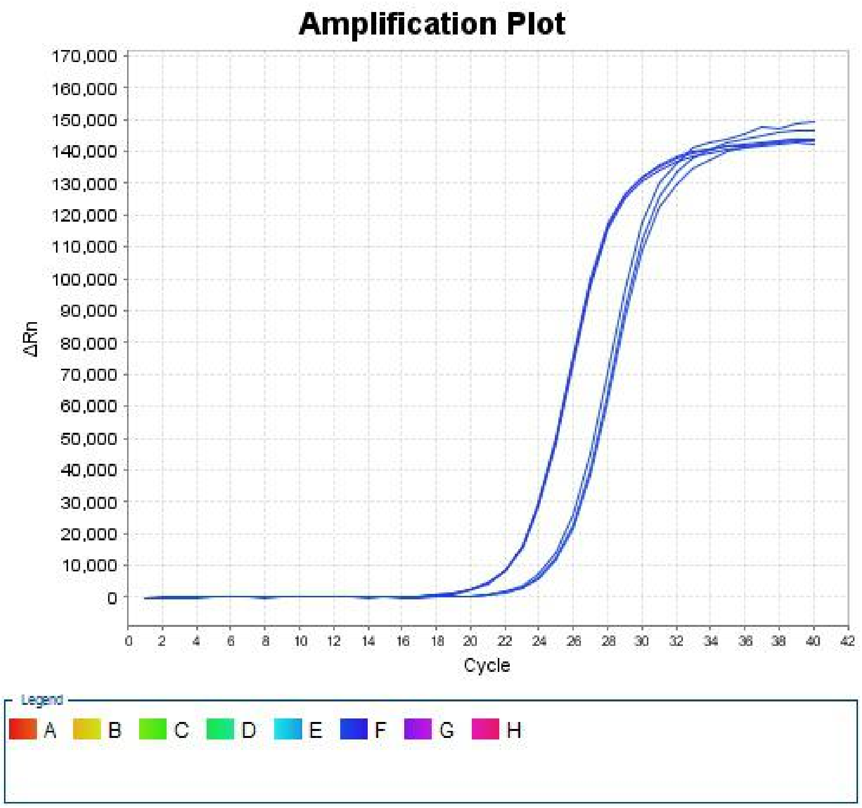

Figure S5

***GRXS5* (At4g15690)**

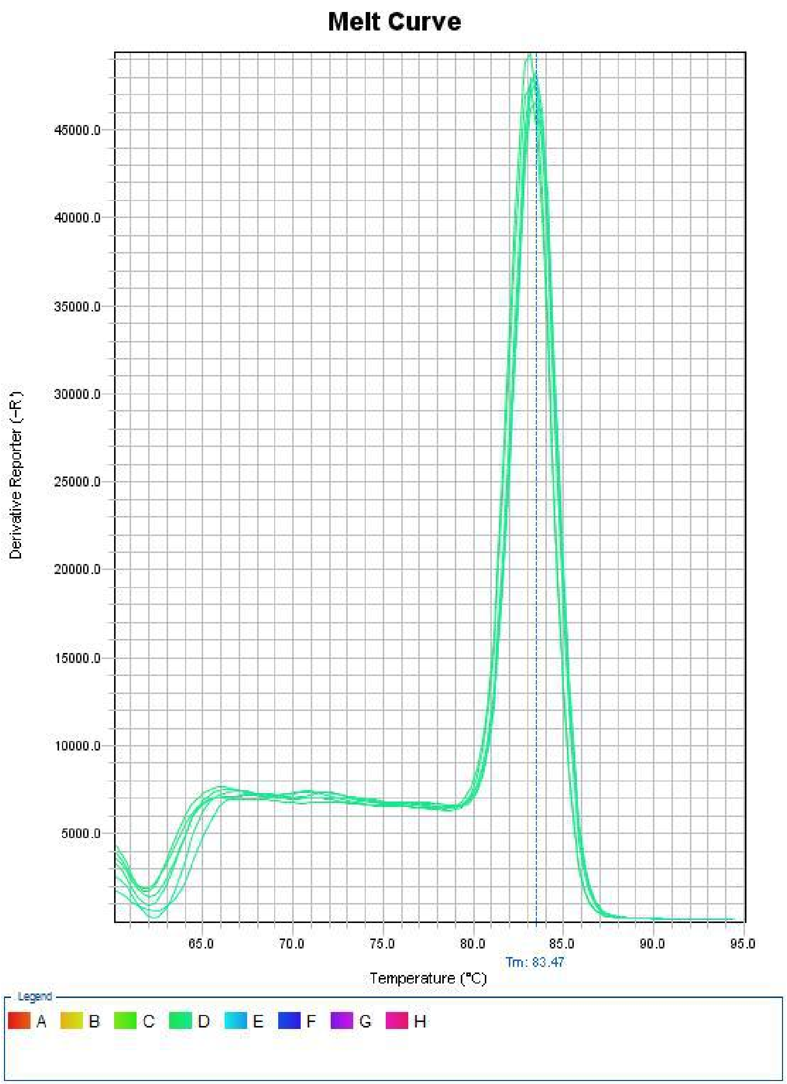

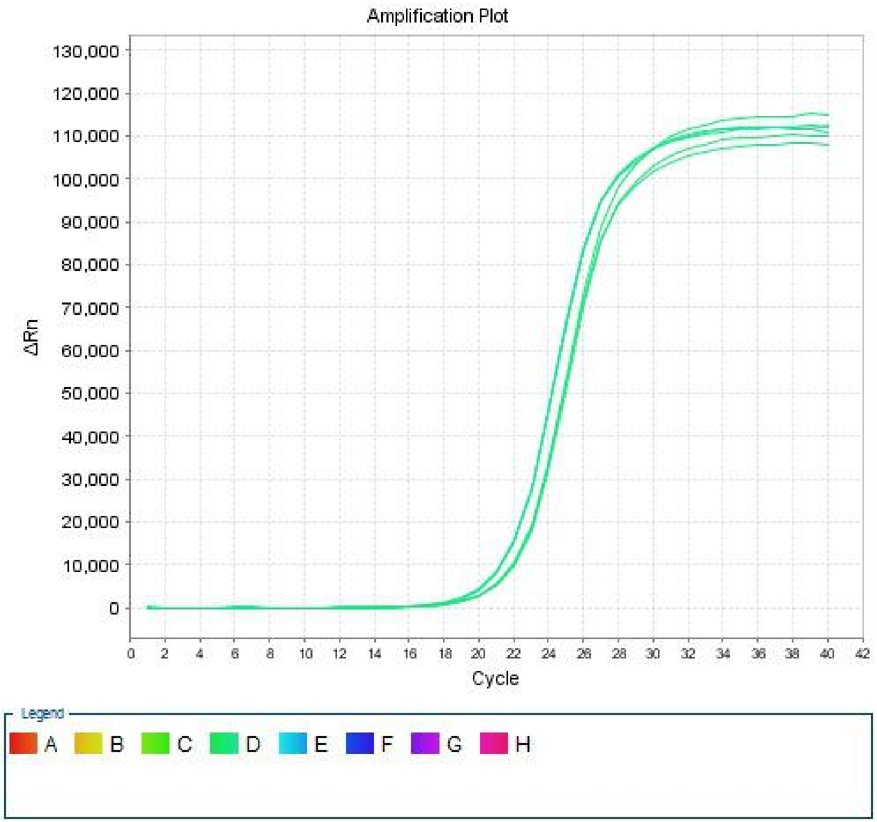

Figure S5

***GRXS7* (At4g15670)**

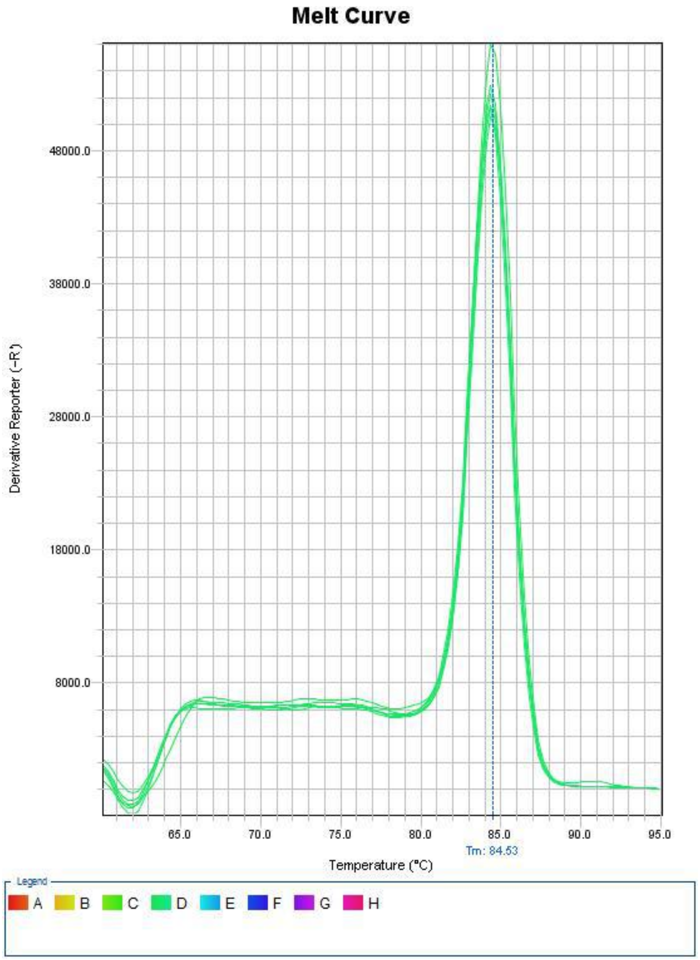

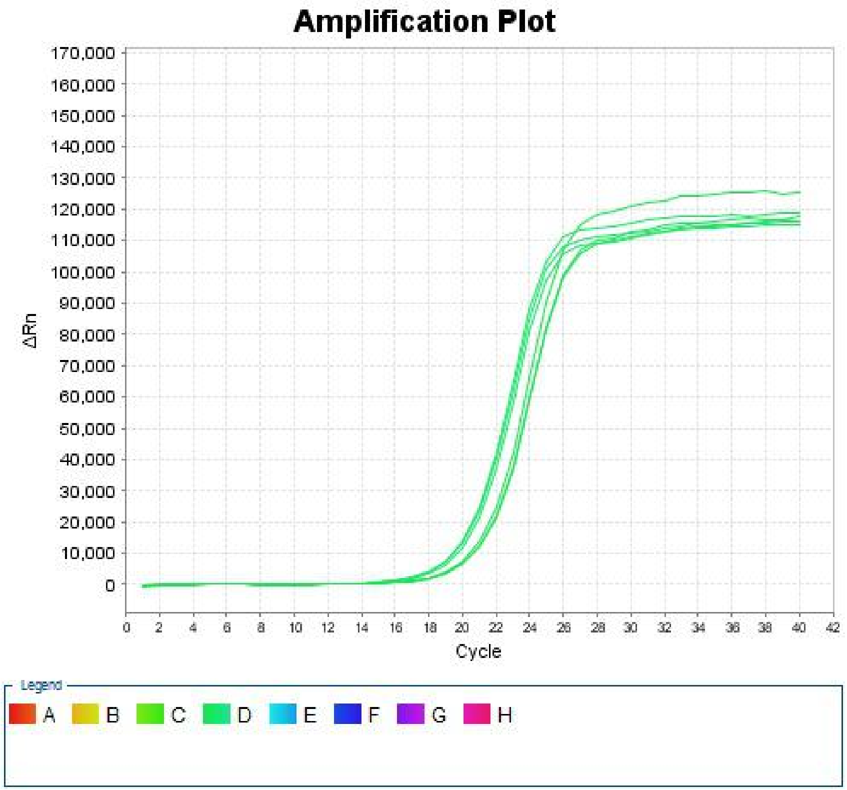

Figure S5

GRXS8 **(At4g15660)**

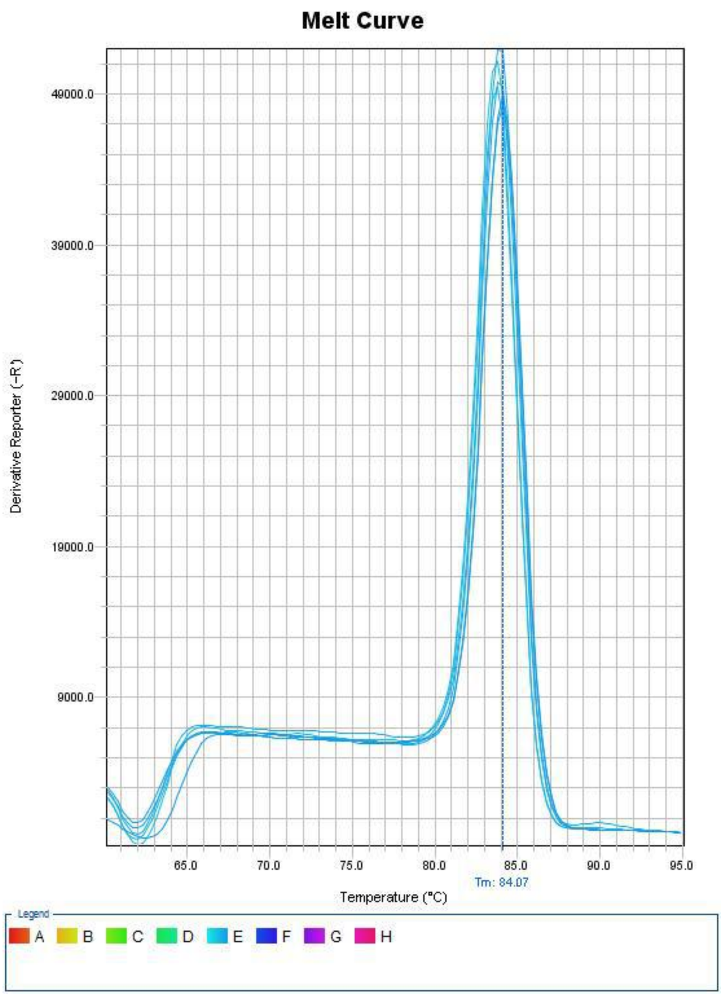

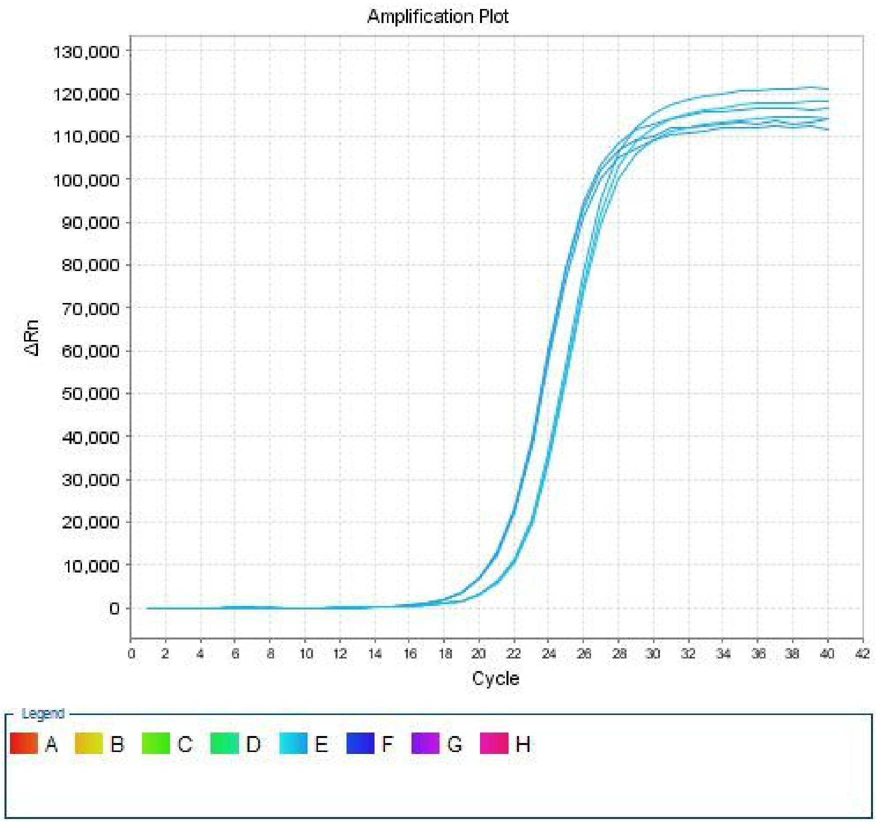

Figure S5

***FD* (At4g35900)**

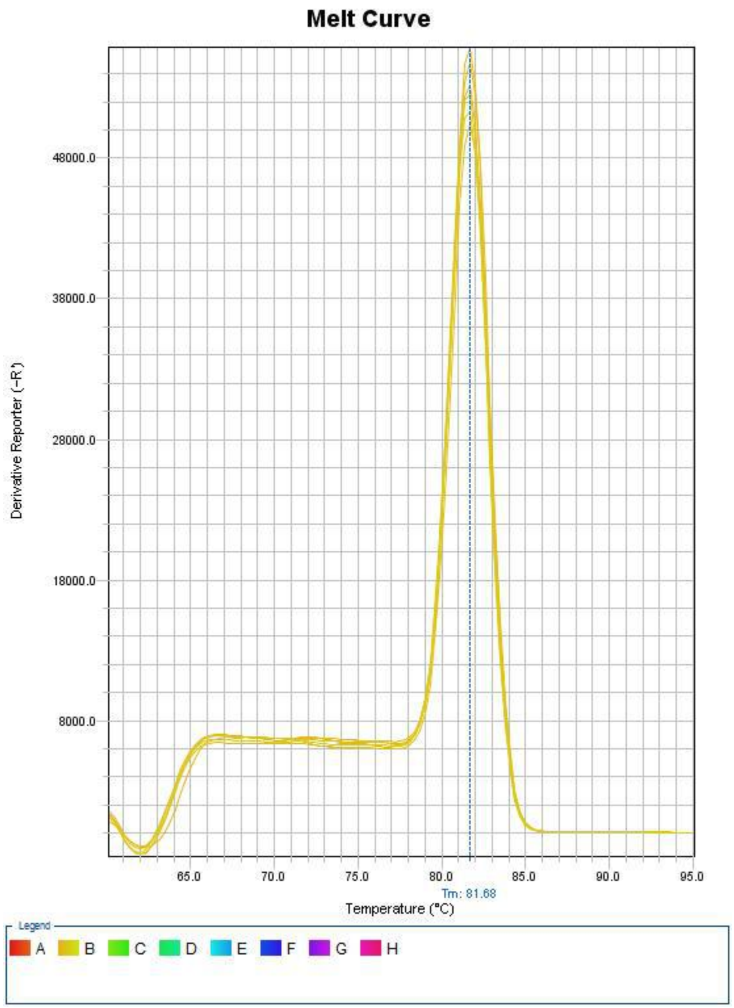

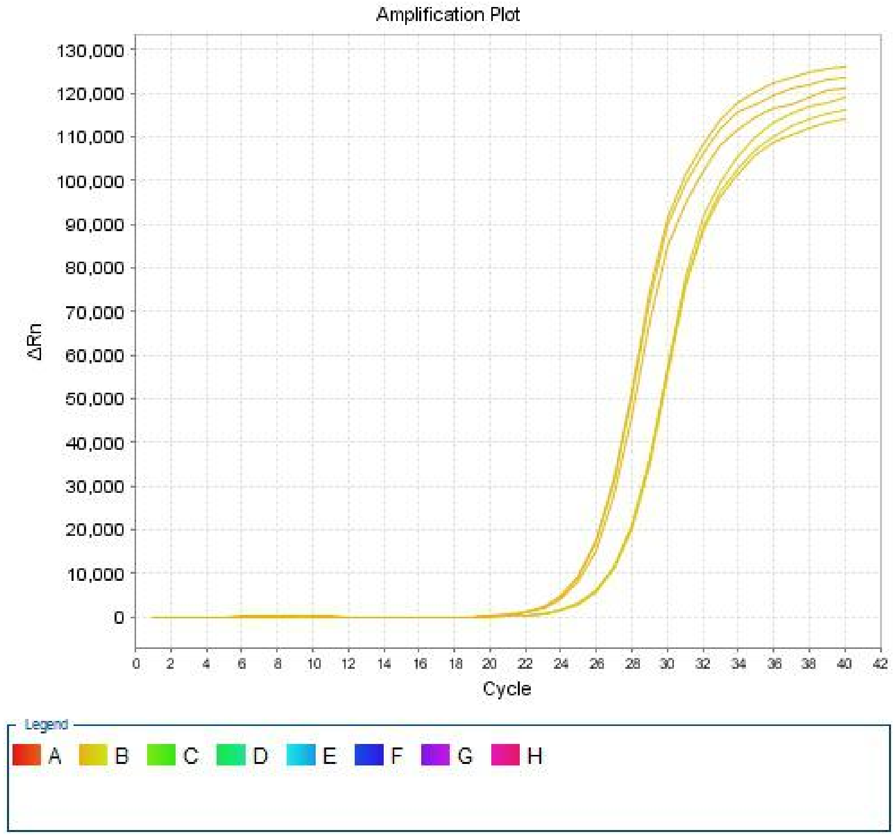

Figure S5

***FT* (At1g65480)**

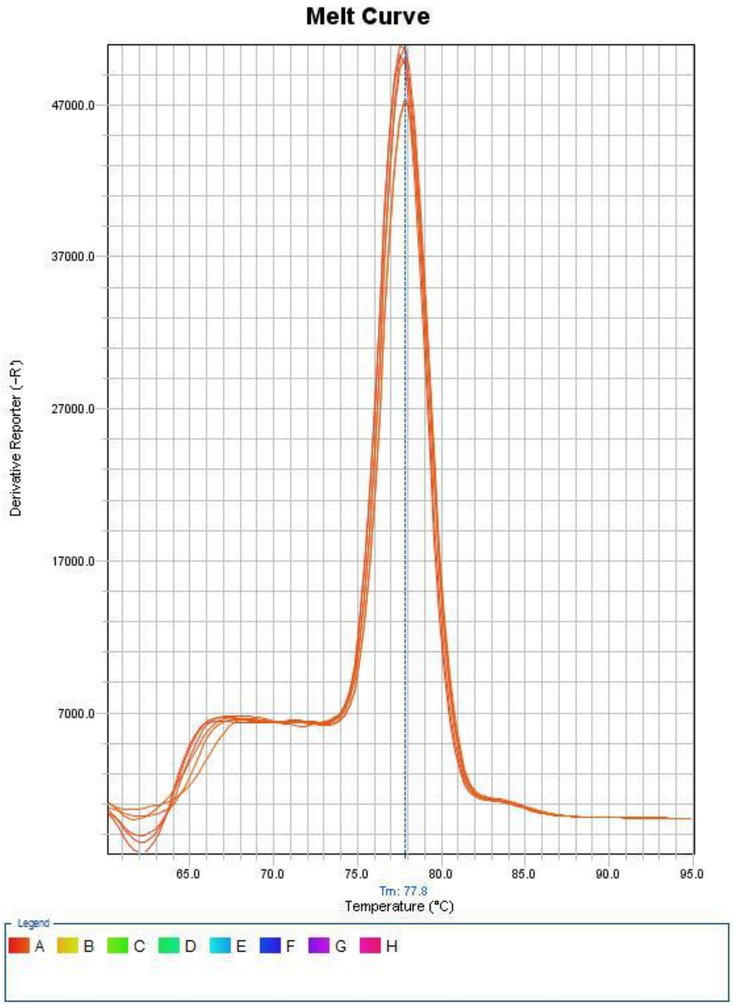

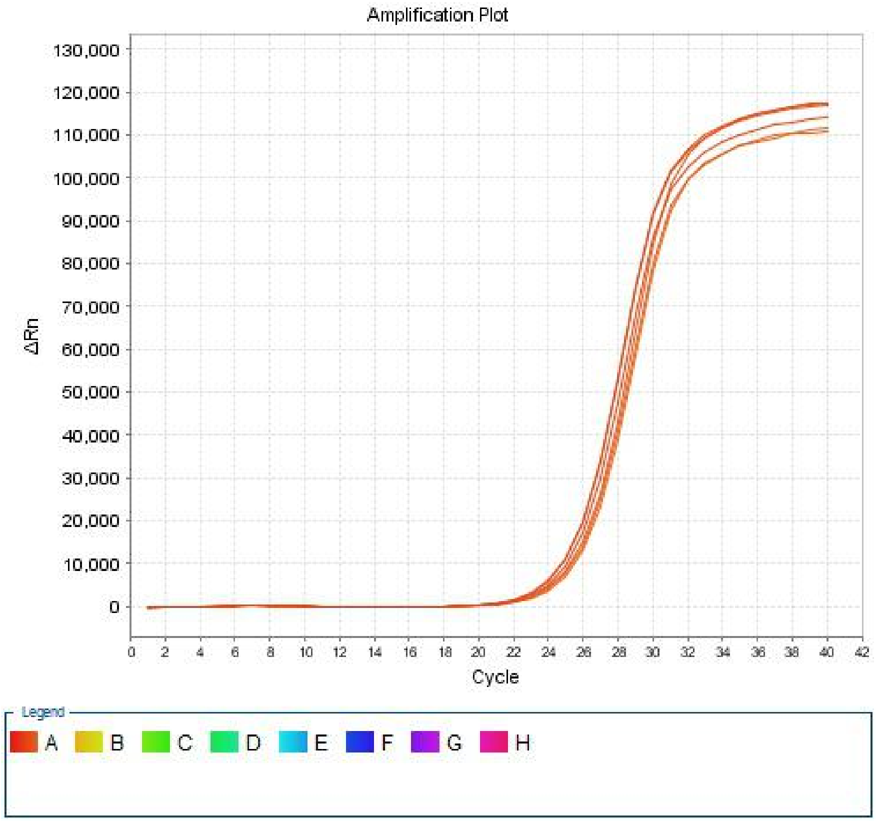

Figure S5

***FUL* (At5g60910)**

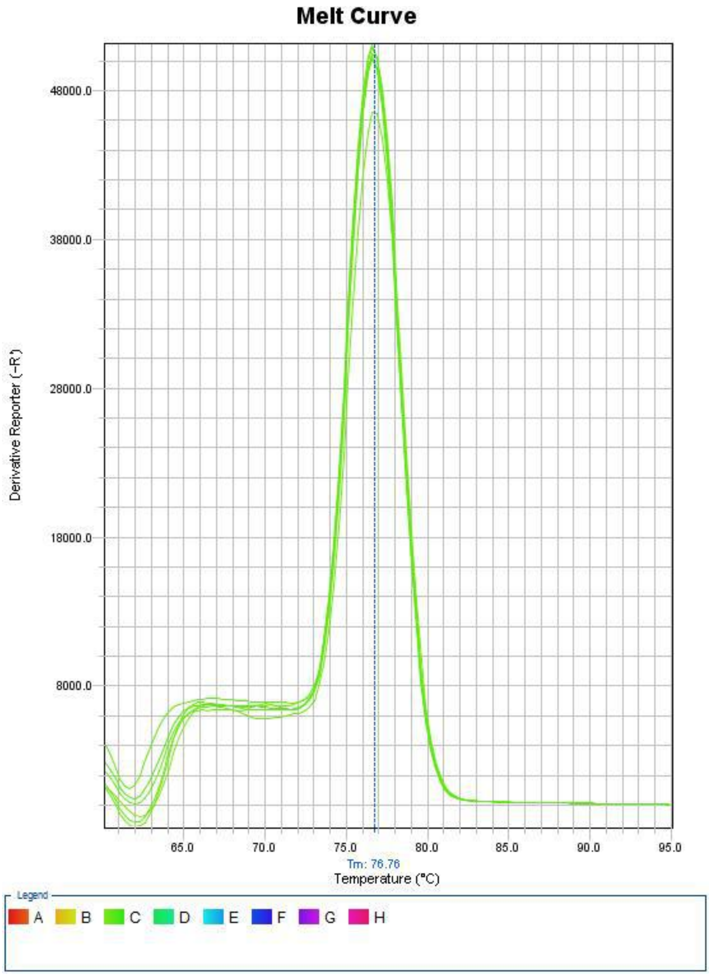

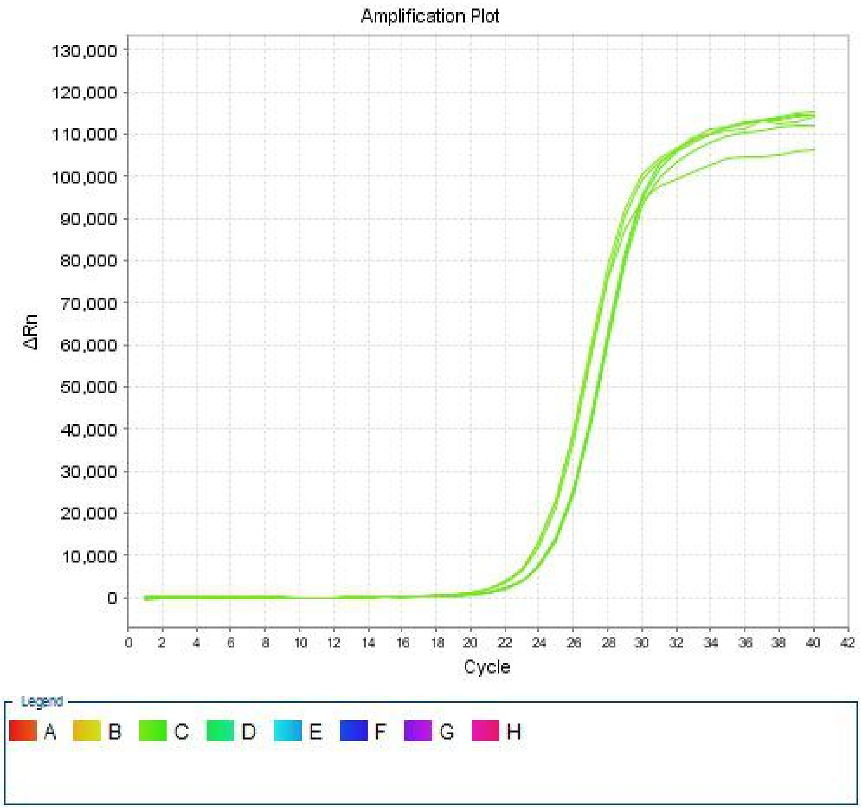

Figure S5

***AtHB-2* (At4g16780)**

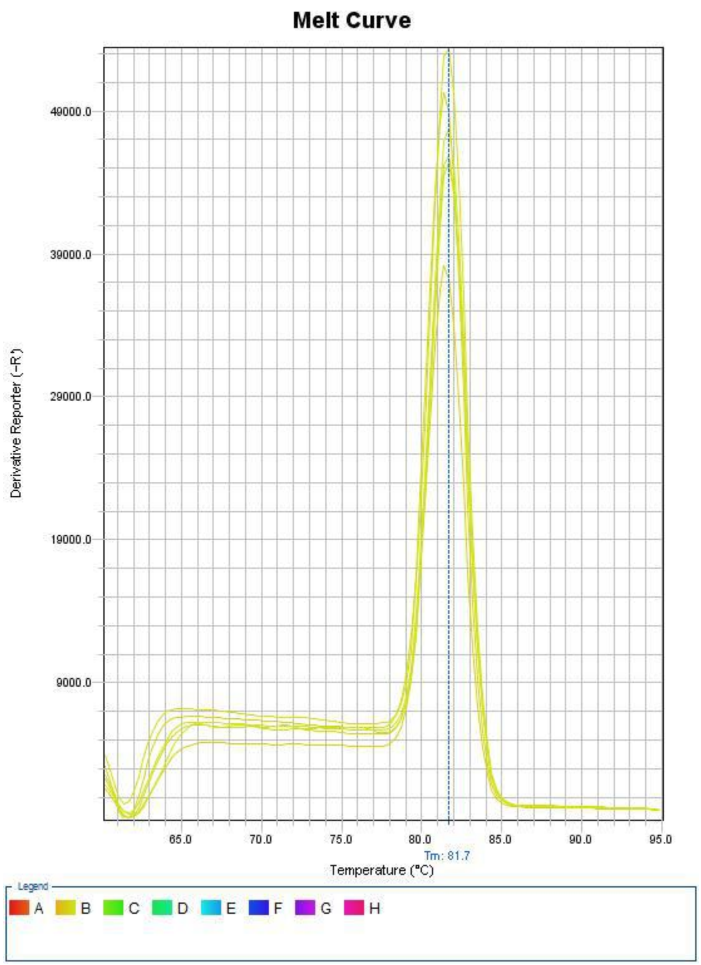

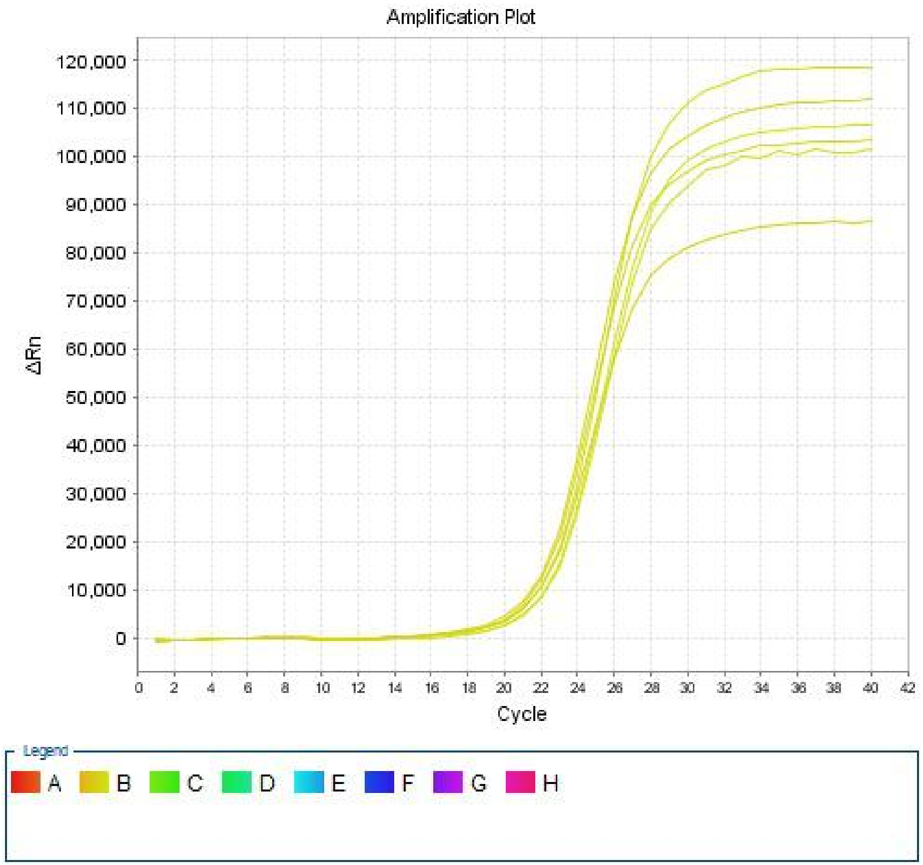

Figure S5

***HFR1* (At1g02340)**

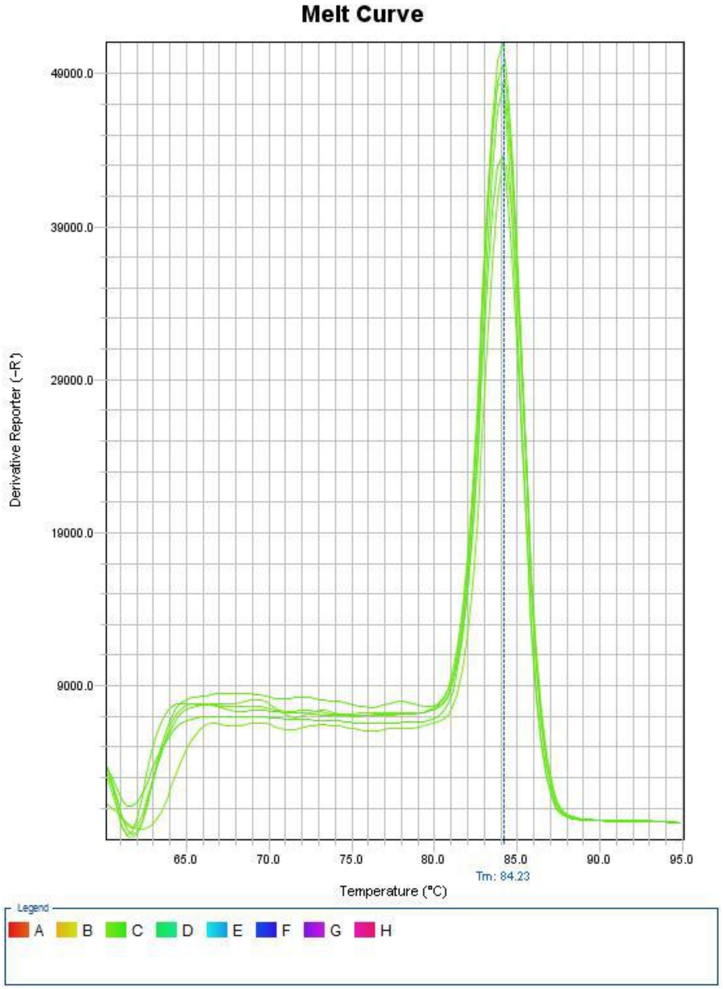

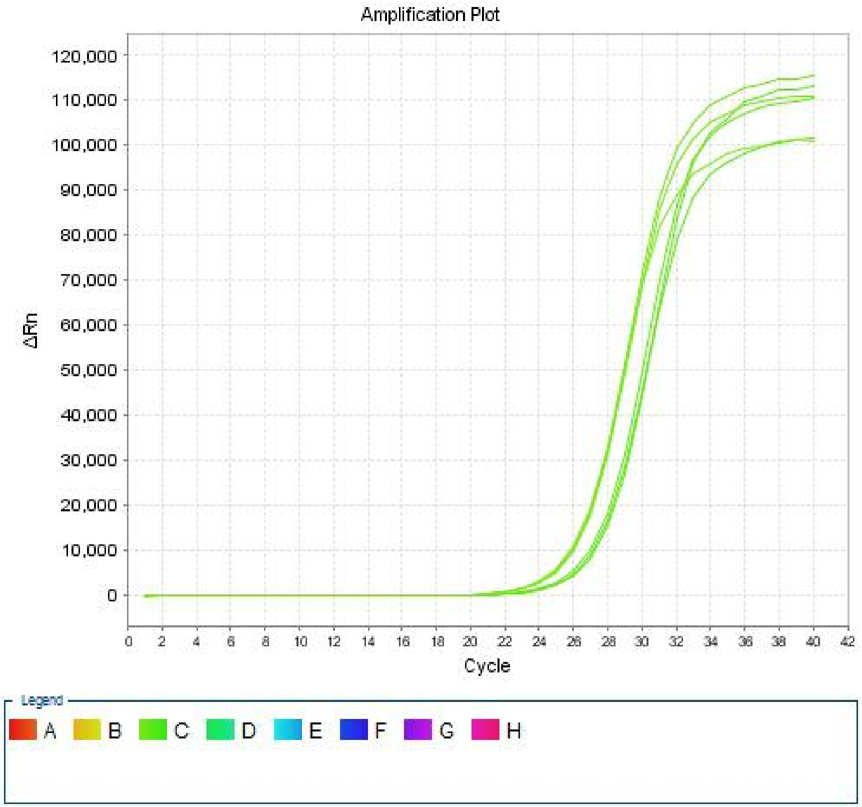

Figure S5

***PIL1* (At2g46970)**

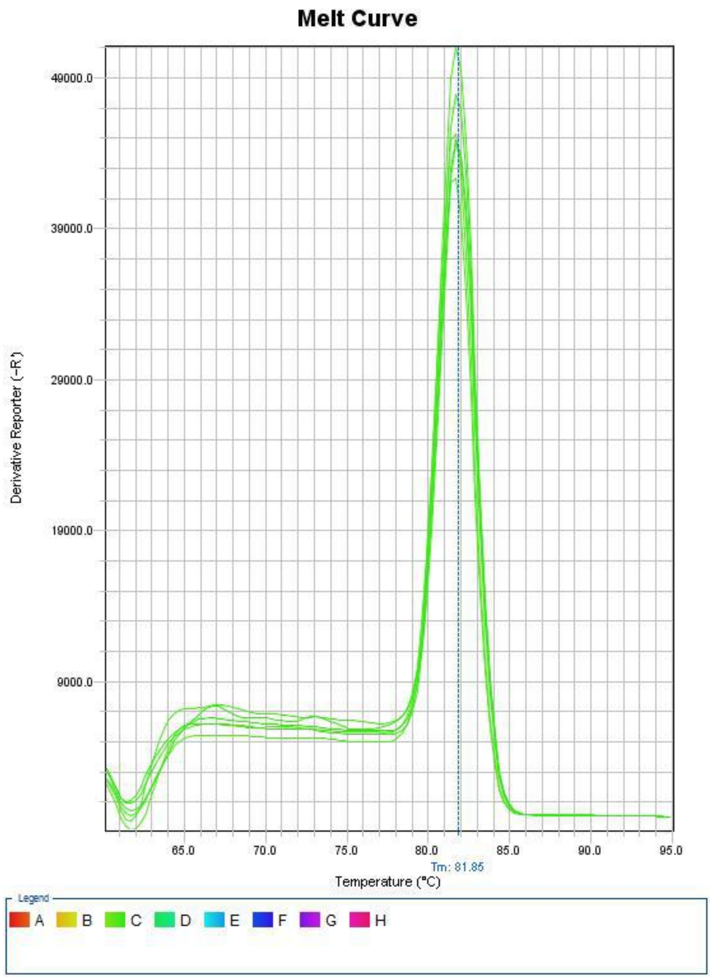

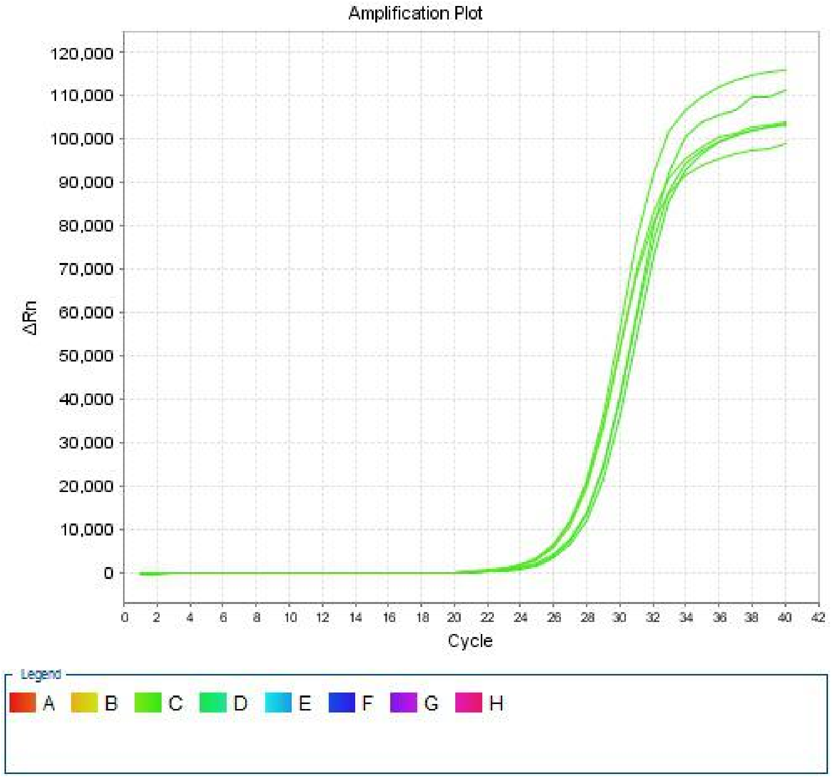

Supplement: FIGURE S5 — Melting curve and amplification plot of all qPCR results. [file Image_5.PDF]
