# Supplementary material for: RNA-Seq Profiling Shows Divergent Gene Expression Patterns in Arabidopsis Grown under Different Densities
Source: Front Plant Sci. 2017 Nov 28;8:2001. doi: 10.3389/fpls.2017.02001 (PMC5712407; doi:10.3389/fpls.2017.02001)
Supplement: TABLE S1 — Summary of the sequencing and assembly. [file Tables_1-4.docx]

**TABLE** **S1.** Summary of the sequencing and assembly.

HD: high density; LD: low density.**TABLE S2.** Summary of the sequencing data filtered by Q20.

HD: high density; LD: low density.**TABLE S3.** Mapped results of the RNA sequencing data.

HD: high density; LD: low density.**TABLE S4.** Detailed analysis of gene map counts.

HD: high density; LD: low density.
