# Supplementary material for: RNA-Seq Profiling Shows Divergent Gene Expression Patterns in Arabidopsis Grown under Different Densities
Source: Front Plant Sci. 2017 Nov 28;8:2001. doi: 10.3389/fpls.2017.02001 (PMC5712407; doi:10.3389/fpls.2017.02001)
Supplement: TABLE S9 — qPCR primers used in this study. [file Table_9.docx]

**TABLE S9.** qPCR primers used in this study.
